# The Involvement of a Polyphenol-Rich Extract of Black Chokeberry in Oxidative Stress on Experimental Arterial Hypertension

**DOI:** 10.1155/2013/912769

**Published:** 2013-02-27

**Authors:** Manuela Ciocoiu, Laurentiu Badescu, Anca Miron, Magda Badescu

**Affiliations:** ^1^Department of Pathophysiology, Faculty of Medicine, University of Medicine and Pharmacy “Grigore T. Popa,” 700115 Iasi, Romania; ^2^Department of Cell and Molecular Biology, Faculty of Medicine, University of Medicine and Pharmacy “Grigore T. Popa,” 700115 Iasi, Romania; ^3^Department of Pharmacognosy, Faculty of Pharmacy, University of Medicine and Pharmacy “Grigore T. Popa,” 700115 Iasi, Romania

## Abstract

The aim of this study is to characterize the content of *Aronia melanocarpa* Elliott (black chokeberry) extract and also to estimate the influence of polyphenolic compounds contained in chokeberries on oxidative stress, on an L-NAME-induced experimental model of arterial hypertension. The rat blood pressure values were recorded using a CODA Noninvasive Blood Pressure System. HPLC/DAD coupled with ElectroSpray Ionization-Mass Spectrometry allowed identification of five phenolic compounds in berries ethanolic extract as follows: chlorogenic acid, kuromanin, rutin, hyperoside, and quercetin. The serous activity of glutathione-peroxidase (GSH-Px) has significantly lower values in the hypertensive (AHT) group as compared to the group protected by polyphenols (AHT + P). The total antioxidant capacity (TAC) values are lower in the AHT group and they are significantly higher in the AHT + P group. All the measured blood pressure components revealed a biostatistically significant blood pressure drop between the AHT group and the AHT + P group. The results reveal the normalization of the reduced glutathion (GSH) concentration as well as a considerable reduction in the malondialdehyde (MDA) serum concentration in the AHT + P group. Ethanolic extract of black chokeberry fruits not only has a potential value as a prophylactic agent but also may function as a nutritional supplement in the management of arterial hypertension.

## 1. Introduction

Hypertension is a significant cardiovascular risk factor, associated to endothelial dysfunction and oxidative stress. The oxidative process participates in increasing systemic arterial pressure, reducing NO availability and vasodilation [1]. Oxidative stress is involved in remodelling the myocardial architecture and as a consequence, in the development of left ventricular hypertrophy [2, 3].

Assessment of antioxidant activities and lipid peroxidation byproducts in hypertensive subjects indicates an excessive amount of ROS and a reduction of antioxidant mechanism activity in both blood as well as in several other cellular systems, including not only vascular wall cells but also those found in circulating blood [4].

Dietary polyphenols are mostly derivatives and/or isomers of flavones, isoflavones, flavonols, catechins, and phenolic acids.* Aronia melanocarpa* Elliot (*Rosaceae*, black chokeberry) is a shrub native to North America. Its berries, which are rich in polyphenols, have been used by native Indians both as a remedy and as a food [5]. Anthocyanins (cyanidin glycosides), flavonoids (quercetin glycosides), chlorogenic acids, and proanthocyanidins are the main polyphenols identified in *Aronia *berries [6]. Several reports indicated that extracts from *Aronia *berries exhibited different biological effects both *in vitro* and *in vivo* (antioxidant, gastroprotective, hepatoprotective, and antiproliferative activities not only via antioxidant pathways, but also via impacting signal transduction/intracellular signalling cascades, impacting apoptosis, etc.) [7, 8].

The aim of this study is to characterize the content of *Aronia melanocarpa* extract and also to estimate the influence of polyphenolic compounds contained in chokeberries on oxidative stress, on an L-NAME induced experimental model of arterial hypertension.

## 2. Materials and Methods

The experimental study fulfils all the requirements of the guide regarding the use of laboratory animals and biological preparations issued by the International Society of Pain Study (IASP) and the European Council Committee (86/609/EEC). Also, the study was evaluated and accepted by the professional ethics committee of Grigore T. Popa University of Medicine and Pharmacy of Iasi (approval no. 9803/12.09.2006).

### 2.1. Preparation of Extract and Chemical Determinations

#### 2.1.1. Chemicals

Folin-Ciocalteu's phenol reagent, chlorogenic acid, and rutin trihydrate were from Merck (Darmstadt, Germany). Quercetin dihydrate, hyperoside, and kuromanin chloride were from Carl Roth (Karlsruhe, Germany). Gallic acid, caffeic acid, and (+)-catechin hydrate were purchased from Sigma-Aldrich (Steinheim, Germany). Except for HPLC grade solvents, all other solvents and reagents were of analytical grade. Ultrapure water was obtained using a SG Water Ultra Clear TWF water purification system (Barsbüttel, Germany).

#### 2.1.2. Plant Material

Ripe berries of *Aronia melanocarpa* Elliott (*Rosaceae*, black chokeberry) were sampled in Botanical Garden, Iasi, Romania. A herbarium voucher sample (AMF.09) is deposited in the Department of Pharmacognosy, School of Pharmacy, University of Medicine and Pharmacy “Grigore T. Popa,” Iasi, Romania. The berries were shade-dried at room temperature for one week. 

#### 2.1.3. Extraction

Dried berries (100 g) were chopped into small pieces and extracted with 3 × 700 mL ethanol using a magnetic stirrer (FALC F30ST), each time for 3 h. The combined extracts were taken to dryness by evaporation under reduced pressure (BÜCHI R-210 Rotavapor, BÜCHI V-850 vacuum controller, and BÜCHI V-700 vacuum pump).

#### 2.1.4. Total Phenolic Content

Total phenolics quantification was performed by Folin-Ciocalteu method [9]. Briefly, berries extract was mixed with 3.16 mL of water and 0.2 mL of Folin-Ciocalteu's phenol reagent. After 5 min., 0.6 mL of 20% sodium carbonate were added. The absorbance was measured at 765 nm after 2 h of incubation at room temperature. A calibration curve was plotted using gallic acid as standard. The total phenolic content was expressed as mg gallic acid equivalents/g extract. Sample was assayed in triplicate and the results were given as the mean ± SD.

#### 2.1.5. Total Anthocyanin Content

Anthocyanins quantification was performed according to a described procedure [10, 11]. Berries extract was mixed with methanol-hydrochloric acid (99 : 1, v : v) and kept at room temperature, in dark, for 2 h followed by centrifugation (1000 ×g, 15 min). Anthocyanin content in supernatant was measured both at 530 nm and 657 nm. Absorbance values were converted into anthocyanin concentration using an extinction coefficient of 31.6 M^−1^ cm^−1^. The results were expressed as *μ*mole anthocyanin/g extract. Sample was assayed in triplicate and the results were given as the mean ± SD. 

#### 2.1.6. HPLC/DAD/ESI-MS Analysis

(High-Performance Liquid Chromatography coupled with Diode Array Detection and ElectroSpray Ionization-Mass Spectrometry) was conducted on an Agilent 1200 Series HPLC system with a diode array detector coupled to an Agilent 6520 Accurate-Mass Q-TOF LC/MS system (Quadrupole Time-of Flight Liquid Chromatography/Mass Spectrometry) equipped with an ESI source. Separations were done on a Zorbax Eclipse XDB-C18 column (150 × 4.6 mm, i.d. 5 *μ*m). The mobile phase consisted of (A) water and acetic acid (99 : 1, v/v) and (B) acetonitrile and acetic acid (99 : 1, v/v). The elution profile was as follows: 0% B (0–3 min, isocratic); 0–7% B in A (3–5 min, linear gradient); 7% B in A (5–20 min, isocratic); 7–10% B in A (20–30 min, linear gradient); 10–15% B in A (30–35 min, linear gradient); 15% B in A (35–50 min, isocratic); 15–20% B in A (50–65 min, linear gradient); 20–30% B in A (65–80 min, linear gradient); 30–40% B in A (80–85 min, linear gradient); 40–100% B (85–100 min, linear gradient); 100% B (100–103 min, isocratic); 100–0% B in A (103–120 min, linear gradient). The flow rate was 0.4 mL/min. Volumes of 20 *μ*L were injected. The compounds were monitored at 254, 280 and 320 nm; anthocyanins were monitored at 515 nm. Mass spectrometric detection was performed in the negative ion mode for nonanthocyanin polyphenols and in the positive mode for anthocyanins. The mass spectrometric conditions for negative and positive ion mode were as follows: drying gas (N_2_) flow rate 7.0 L/min; drying gas temperature 220°C; nebuliser pressure 15 psig. In negative ion mode the capillary voltage was set to −4.2 kV and the skimmer voltage to −60 V. In positive ion mode the capillary voltage was set to 4.2 kV and the skimmer voltage to 60 V. A fragmentor voltage of 200 V was used in both modes. The full-scan mass spectra of the investigated compounds were acquired in the range 50–2000 *m/z* [6]. Data were collected and processed using a MassHunter Workstation software.

### 2.2. Animal Treatments and Biochemical Determinations

The dry polyphenol extract was diluted in DMSO, 100 mL polyphenolic solution containing 840 mg natural polyphenols, 95 mL distilled water, and 5 mL DMSO. After repeated testing, it was found that the dose of polyphenols extracted from the fruits of *Aronia melanocarpa* to be administered as enteral solution (by tube feeding) is 0.050 g/kg body every two days. The experiment used active therapeutic doses, well-determined fractions of DL50 on an experimental model of arterial hypertension. Fractions of DL50 are doses representing 1/5, 1/10, 1/20, and 1/40 of DL50. The dose representing 1/20 of DL50 was chosen, as it is the smallest dose that determined the pharmacodynamic effect that is being researched, without producing significant toxic effects.

The research was performed on Wistar white rats, with an average weight of 250–280 g, which were divided into 4 groups of 12, namely: (i) Group W—control, normal animals, that did not receive natural polyphenols; (ii) Group AHT—animals that were administered L-NAME 40 mg/kg body/day, i.p., at every 2 days, for 8 weeks; (iii) Group P—animals that were administered polyphenols under the form of solution, from the extract obtained from the *Aronia melanocarpa* fruit, at every 2 days, for 8 weeks; (iv) Group AHT + P—animals that were administered polyphenols in the dosage mentioned p.o. at every 2 days, concomitantly with L-NAME, for 8 weeks.

The blood samples necessary to the biochemical determinations were drawn from the retrorbital venous sinus. *The malondialdehyde (MDA) concentration*—the index of lipid peroxidation—was determined by the Ohkawa method using the thiobarbituric acid [12]. The MDA concentration was expressed in nmol/mL. *Glutathione peroxidase (GSH-Px)* (H_2_O_2_ *: GSH oxidoreductase*) was determined by the Gross and Beutler method [13]; the GSH-Px activity was expressed in *μ*M oxidized GSH per minute/g Hb or mg protein. *Reduced glutathione (GSH)* was also determined by the Beutler method, through the use of 5,5′ dithio-bisnitro-benzoic acid (DTNB), and was expressed in *μ*g GSH/mg protein or g Hb in erythrocyte. For the extracellular response the *total antioxidant capacity (TAC)* was determined by using a RANDOX kit for manual use by Randox Laboratories Ltd. The major advantage of this test is to measure the antioxidant capacity of all antioxidants in a biological sample and not just the antioxidant capacity of a single compound. The method is based on formation of the ABTS•+ cation (2,2′-azinobis (3-ethylbenzothiazoline-6-sulfonic acid)) and its scavenging by antioxidant sample constituents (e.g., serum or food) measured by spectrophotometry (decay of green/blue chromophore absorbance is inversely associated with antioxidant sample content and the control antioxidant is Trolox, a hydrophilic vitamin E analog).

The rat *blood pressure* values were recorded using a CODA Non-invasive Blood Pressure System, purchased from Kent Scientific Corporation, which uses a noninvasive blood pressure measuring method. It records the blood volume-pressure by a band attached to the tail, homologated by Bland-Altman testing [14], designed to reveal conformity with an invasive method (radiotelemetry), which enjoys proven accuracy yet is difficult to use in our study. The method is also recommended by the American Heart Association in its blood pressure measuring guide for laboratory animals [15]. The experiment consists in performing at least 6 blood pressure measurements in each laboratory animal, and data collection is by means of the CODA. 

### 2.3. Statistical Data Interpretation

All the data are shown as mean value ± standard error of the mean (SEM). In order to assess the normal distribution of the groups, Shapiro-Wilk test was performed. Additionally, Levene test was performed to confirm the homoscedasticity of the groups, followed by ANOVA and paired or unpaired *t*-test to reveal the pairs of groups that differ biostatistically significantly in term of means. Statistical data interpretation considered the corresponding differences for a given significance threshold: *P* > 0.05: statistically insignificant; *P* < 0.05: statistically significant; *P* < 0.01: strong statistical significance; *P* < 0.001 very strong statistical significance.

## 3. Results

### 3.1. Extraction

Ethanol extraction of black chokeberry fruits yielded 47.17 g extract. The extract was stored at −20°C until used.

### 3.2. Phenolic Contents

Berries ethanolic extract contained 24.87 ± 0.54 mg total phenolics/g and 4.46 ± 0.06 *μ*mole anthocyanin/g.

### 3.3. HPLC/DAD/ESI-MS Analysis

**Figure 1:**
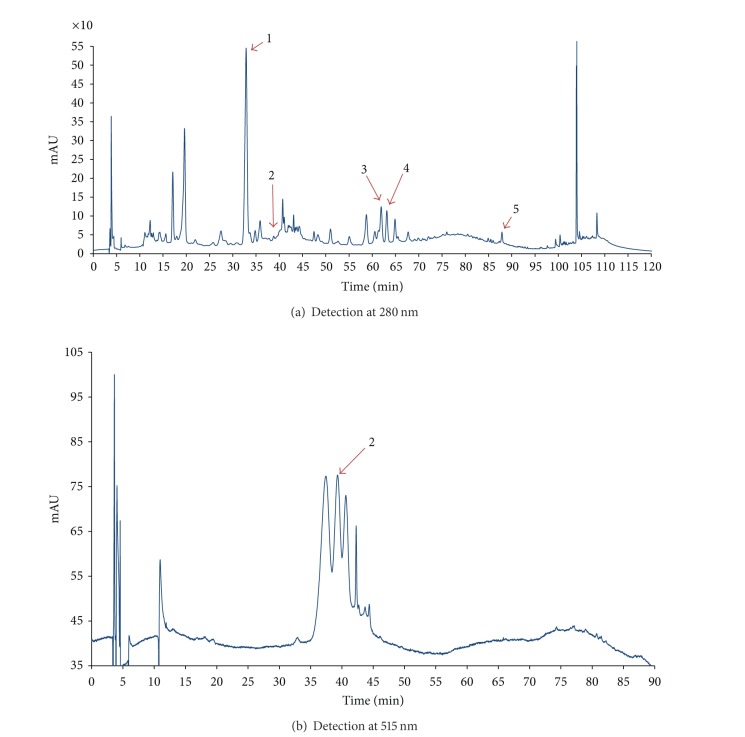
HPLC-DAD chromatograms of ethanolic extract of black chokeberry fruits (1—chlorogenic acid, 2—kuromanin, 3—rutin + unknown compound, 4—hyperoside, and 5—quercetin).

In the present study, the phenolic profile of berries ethanolic extract was characterized by HPLC/DAD/ESI-MS (Figure 1).

HPLC/DAD/ESI-MS allowed identification of chlorogenic acids, quercetin and cyanidin glycosides, and proanthocyanidins as major polyphenols in black chokeberry fruits [16]. (+)-Catechin hydrate, chlorogenic acid, caffeic acid, rutin trihydrate, hyperoside, quercetin dehydrate, and kuromanin chloride were used as standards. Main phenolic constituents were identified by comparison of their retention times and mass spectral data to those of authentic standards. Five phenolic compounds have been detected in berries ethanolic extract as follows: chlorogenic acid, kuromanin, rutin, hyperoside and quercetin; their retention times and mass spectral data are given in Table 1.

### 3.4. GSH-Px Serum Activity in Hypertensive Rats (AHT)

The GSH-Px serum activity in hypertensive rats (AHT) has significantly low values (*P* < 0.001) when compared to the rats in groups W and AHT + P, which is a consequence of oxidative stress increase (Table 2).

As a consequence of oxidative stress increase in hypertensive rats (AHT), the reduced glutathione (GSH) values are significantly low (*P* < 0.001) as compared to the rats in the W and AHT + P groups. The group of hypertensive rats protected by the administration of polyphenolic extract showed significantly higher (*P* < 0.01) values of GSH serum concentration compared to the hypertensive group.

The results achieved reveal a significant serum antioxidant capacity improvement (*P* < 0.001) in the AHT + P rats, the normalization of the GSH concentration, and a considerable reduction in the MDA serum concentration, causing a significant lipid peroxide diminution in the serum. Depending on the significance threshold values (*P*), the statistical analysis of the MDA values reveals significant differences (*P* < 0.01) between the AHT + P and the AHT groups, respectively, and highly significant differences (*P* < 0.001) between the AHT and the W groups.

The TAC levels were significantly decreased in AHT group (*P* < 0.001) as compared to the rats in the W and AHT + P groups. As expected, severe oxidative stress disturbs the antioxidant balance by generating reactive species and decreasing the total antioxidant capacity in the extracellular space. There are similar TAC values in group W and group P and the differences between the AHT and AHT + P groups are statistically significant (*P* < 0.01).

**Figure 2:**
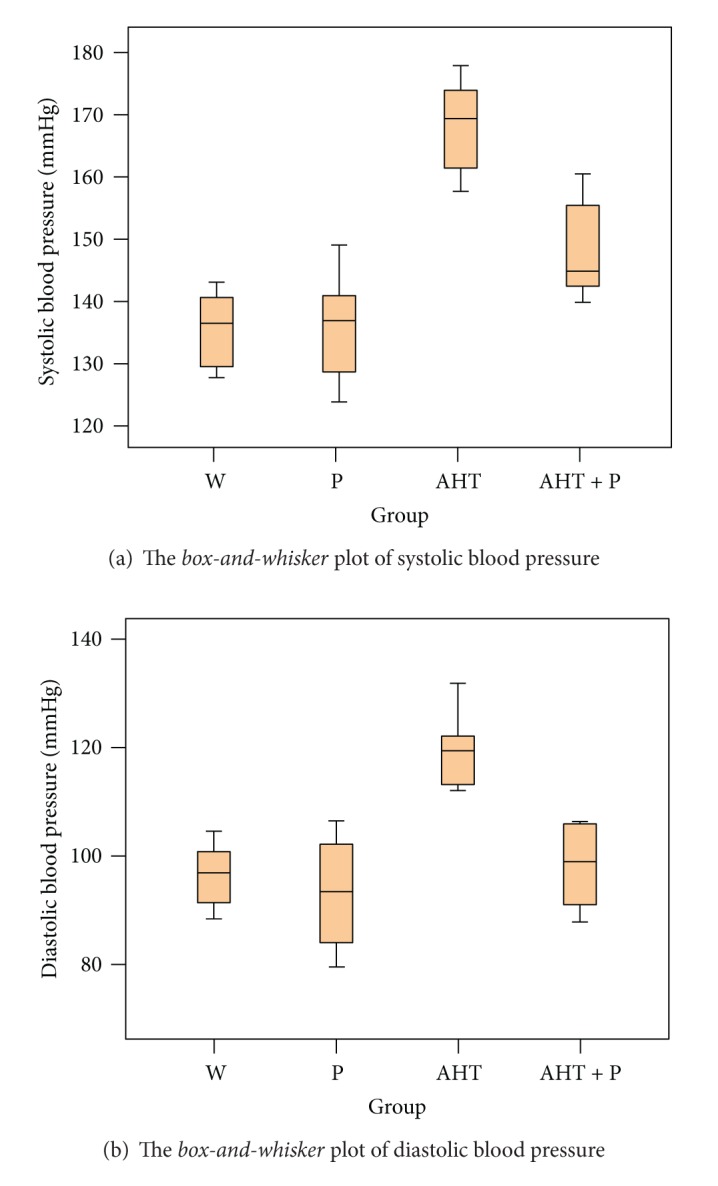
Systolic and diastolic blood pressure at studied groups.

The systolic and diastolic blood pressures, as well as their calculated mean, were measured. *The Shapiro-Wilk test* was positive, which supports sample normality, and the descriptive statistics and box-and-whisker plots are shown in Figure 2.

*The Levene test* confirmed group homoscedasticity, whereas the *ANOVA* test revealed a significant difference between the means of the 4 groups, as concerns systolic and diastolic blood pressure (Table 3). All the measured blood pressure components revealed a biostatistically significant (*P* < 0.05) blood pressure drop between the AHT and the AHT + P groups.

## 4. Discussion

Polyphenols act as free radicals scavengers by donating hydrogen atoms or electrons from phenolic hydroxyls. This is the main mechanism by which polyphenols scavenge many ROS (superoxide anion radical, hydroxyl radical). 

During arterial hypertension, due to the high oxygen consumption, the reactive oxygen species act chiefly on unsaturated lipids, belonging to the membrane, with the formation of certain peroxidation products, which generate MDA. Since the most reactive radicals are short-lived they might be expected to react close to the site where they are formed. Polyphenols vary strongly in their absorption and distribution. They show high affinity for different structures and may therefore be able to decrease oxidative damage mainly at such particular sites. A clear idea about the antioxidant potentials and the dependence of antioxidant activities on the quality and the quantity of phenolic substances can be obtained by comparison of the antioxidant activities of different phenolic extracts at equal total phenol concentrations. 

Recent studies have revealed a total phenolic content of 20–90 mg/kg fresh wt (or mg/L) in strawberry, of 20–40 mg/kg fresh wt (or mg/L) in apple, and of 15–40 in black grape [17]. The results of this study show that the total phenolic content in the black chokeberry extract is similar to that found in fresh strawberries, apples, and black grapes.

In normal conditions, the intrinsic antioxidant systems counteract the effects of oxidative stress. Therefore, the polyphenolic extract used contributes to actively maintain the effects of these systems (this can be construed as an explanation to why the MDA values in the animals from P group are lowered less than the ones in AHT + P group).

Free radicals scavenging activity and metal chelation partially explain polyphenols inhibitory effects on lipid peroxidation and LDL oxidation [18]. In addition, some polyphenols increase the activity of several endogenous antioxidant defence systems (GSH-Px, superoxide dismutase (SOD), and catalase (CAT)) and induce a significant increase in GSH level [19].

Phenolic compounds detected in ethanolic extract of black chokeberry fruits have shown antioxidant effects. Chlorogenic acid, quercetin, and cyanidin are effective radical scavengers and iron chelators; glycosylated derivatives of quercetin and cyanidin have a lower scavenging activity in comparison to quercetin and cyanidin, respectively [20].

However, it may still be true that specific antioxidants are preventive, and since polyphenols are both potent antioxidants and abundant in plant foods they are likely candidates for fulfilment of the antioxidant hypothesis.

The current study revealed an important reduction in the antioxidant mechanisms, both in GSH levels and antioxidant enzymatic activities in the hypertensive group. Reduced levels of GSH have been related to an extensive number of metabolic and gene expression disturbances, since the tripeptide is not only an efficient antioxidant but also an important regulatory substance in biological systems. The low activity of GSH is a consequence and not a cause of the increase in the oxidative status. Reactive oxygen species oxidized GSH to GSSG, leading to a decrease in GSH and an increase in GSSG concentrations. Long-time oxidative stress can consume antioxidants and reduce SOD, CAT, and GSH-Px levels in cardiovascular diseases in general, and, especially, in arterial hypertension.

A wide range of evidence suggests that the Keap1 (Kelch ECH associating protein)-Nrf2 (nuclear-factor-erythroid 2-related factor) complex constitutes a sensor of oxidative stress involved in triggering antioxidant-response-element- (ARE-) mediated gene expression to restore the cellular redox status [21]. Under basal conditions, Nrf2 interacts with a cytosolic repressor protein Keap1 limiting Nrf2 mediated gene expression [22]. In cells exposed to oxidative stress, Nrf2 is released from Keap1 and translocates to the nucleus, where it activates ARE dependent transcription of phase II and antioxidant defense enzymes, such as NADPH:quinone oxidoreductase, glutathione-S transferase, glutathione peroxidase, and heme oxygenase-1 [23]. Polyphenols may modify the capability of Keap1 to seclude Nrf2 and/or activate MAPK proteins (ERK, JNK and p38). Polyphenols could also be involved in Nrf2 stabilization [24].

The role of antioxidant nutrients in fighting against oxidative stress is well established in several diseases including cardiovascular and neurological pathologies [25]. In this sense, researchers had materially proved that following consumption of diets rich in fruits and vegetables there was an increase in serum TAC [26]. The decline of MDA levels may be caused by increased antioxidant status. 

The blood pressure decrease effect of polyphenols could be due to particular actions, nonmediated by estrogenic receptors, of nitric oxide or superoxide anion bio-availability modulation by polyphenols [27, 28]. The significant blood pressure value drop in the hypertension group protected by polyphenols could be related to their ability to decrease *in vivo *reactive oxygen species production. In our study, polyphenols intake is associated with blood pressure decrease, not through the lowering of heart rate, but through the antioxidant mechanism especially.

The TAC levels were significantly decreased in the hypertensive group. In this experimental model, all groups were put on the same diet. The reduction in antioxidant mechanisms can be neutralized through natural polyphenol compounds of *Aronia melanocarpa* which will maintain TAC to levels capable of neutralizing ROS effects. The decay in TAC, as significant as it was, was not as severe as the changes observed in intracellular enzyme activity. 

The TAC evaluation, used with other oxidative stress and antioxidant defense biomarkers, constitutes the first step in search for a healthy body status. In order to form strategies for the intervention and prevention of cardiovascular diseases, an understanding of the basic molecular mechanisms by prophylactic agents is required. 

The study demonstrated that an ethanolic extract from fruits of *Aronia melanocarpa* Elliott is able to reduce endothelial dysfunction and improve total antioxidant capacity in early arterial hypertension. Evidence shows that polyphenols can increase the antioxidative capacity of plasma and that this effect is directly related to the plasma concentration and the intrinsic antioxidant capacity of the compounds. Evidence that the intake of catechins and related procyanidins are linked to the increased antioxidant capacity of plasma is necessary in order to assess more accurately whether there is a relationship between polyphenol intakes and health risks mediated by antioxidation.

A good way to raise the intake of antioxidants from *Aronia melanocarpa* fruits is to increase the proportion of consumption, and another effective way is to substitute the fruit and vegetables that have low antioxidant capacity with antioxidant-rich extract from *Aronia* fruits. In addition, a colorful variety of all fruit and vegetables, healthfully prepared, makes a significant contribution to a diet that promotes good health.

There are potential clinical benefits in using the polyphenolic extract coupled with the antihypertensive drugs with therapeutic purposes. This would lead to using a smaller dose of antihypertensive drugs and thus diminishing the secondary effects they produce.

## 5. Conclusions

Ethanolic extract of black chokeberry fruits has a potential value as prophylactic agent, but also may function as a nutritional supplement in the therapy of arterial hypertension. The role of the polyphenolic extract of *Aronia melanocarpa* is to prevent the total antioxidant capacity decrease and also to reduce the oxidative stress. Knowing the cellular and molecular mechanisms through which each compound of the *Aronia melanocarpa* extract acts in arterial hypertension requires further studies.

## Figures and Tables

**Table 1:** Retention time and mass spectral data of polyphenolic compounds detected in ethanolic extract of black chokeberry fruits.

Nonanthocyanin polyphenols				
Peak no.	Rt (min)	Mass spectral data		Peak assignment
		Deprotonated molecule [M–H]^−^ (*m/z*)	Fragment ions (*m/z*)	

1	32.9	352.90	190.93 [M-H-Caffeoyl]	Chlorogenic acid (5-O-caffeoyl quinic acid)
3	61.9	608.87	—	Rutin* (quercetin-3-O-glucorhamnoside)
4	63.2	462.86	—	Hyperoside (quercetin-3-O-galactoside)
5	87.9	300.86	—	Quercetin

Anthocyanins				

Peak no.	Rt (min)	Mass spectral data		Peak assignment
		Molecular ion [M]^+^ (*m/z*)	Fragment ions (*m/z*)	

2	39.3	449.21	287.13 [M-Glucose]	Kuromanin (cyanidin-3-O-glucoside)

*Rutin coeluted with a compound (462.86 *m*/*z*), possibly another quercetin glycoside.

Rt: retention time.

**Table 2:** GSH-Px, GSH, and TAC modifications in the studied groups.

	W	P	AHT	AHT + P
MDA (nmol/mL)	0	0	8.76 × 10^−2^***	6.43 × 10^−2^^##^
GSH-Px (*μ*mol/mL)	2.53 ± 0.19	2.37 ± 0.49*	1.17 ± 0.20***	1.56 ± 0.21^##^
GSH (*μ*mol/mL)	7.29 ± 0.21	7.53 ± 0.40*	5.10 ± 0.49***	6.71 ± 0.35^##^
TAC (mmol/L)	1.55 ± 0.29	1.58 ± 0.31	1.31 ± 0.16**	1.53 ± 0.27^##^

Values are mean ± SEM (*n* = 12 animals). Statistical analyses:

*P < 0.05; **P < 0.01; ***P < 0.001, versus W group.

^#^ *P* < 0.05; ^##^ *P* < 0.01; ^###^ *P* < 0.001, versus AHT group.

**Table 3:** ANOVA test.

Blood pressure	*F* value	P* value
Systolic	22.901	0.001
Diastolic	13.199	0.001
Mean	16.970	0.001

*P < 0.05 indicates biostatistically significance.
